# CaMKII regulates neuromuscular activity and survival of the human blood fluke *Schistosoma mansoni*

**DOI:** 10.1038/s41598-022-23962-8

**Published:** 2022-11-18

**Authors:** Natasha L. Hirst, Scott P. Lawton, Anthony J. Walker

**Affiliations:** 1 Molecular Parasitology Laboratory, School of Life Sciences Pharmacy and Chemistry, Kingston University, Penrhyn Road, Kingston Upon Thames, KT1 2EE UK; 2 Epidemiology Research Unit, Department of Veterinary and Animal Sciences, Scotland’s Rural College (SRUC), An Lòchran, 10 Inverness Campus, Inverness, IV2 5NA UK

**Keywords:** Parasite biology, Parasite physiology, Kinases

## Abstract

Calcium/calmodulin dependant protein kinase II (CaMKII), an important transducer of Ca^2+^ signals, orchestrates multiple cellular functions in animals. Here we investigated the importance of CaMKII to *Schistosoma mansoni*, a blood parasite that causes human schistosomiasis. We demonstrate that phosphorylated (activated) CaMKII is present in cercariae, schistosomula and adult worms, and show that striking activation occurs in the nervous tissue of these parasite life-stages; CaMKII was also activated in the tegument and muscles of adult worms and the vitellaria of females. Exposure of worms to the anti-schistosomal drug praziquantel (PZQ) induced significant CaMKII activation and depletion of CaMKII protein/activation in adult worms resulted in hypokinesia, reduced vitality and death. At medium confidence (global score ≥ 0.40), *S. mansoni* CaMKII was predicted to interact with 51 proteins, with many containing CaMKII phosphorylation sites and nine mapped to phosphoproteome data including sites within a ryanodine receptor. The CaMKII network was functionally enriched with mitogen-activated protein kinase, Wnt, and notch pathways, and ion-transport and voltage-dependent channel protein domains. Collectively, these data highlight the intricacies of CaMKII signalling in *S. mansoni*, show CaMKII to be an active player in the PZQ-mediated response of schistosomes and highlight CaMKII as a possible target for the development of novel anti-schistosome therapeutics.

## Introduction

*Schistosoma mansoni* is one of three major human-infective schistosome species that cause human schistosomiasis in almost 240 million people globally^1,2^. Schistosome parasites infect humans when cercariae, released from infected freshwater snails, are attracted to skin and penetrate^3^. The *S. mansoni* cercariae then rapidly transform into schistosomula which invade the vasculature and migrate in the blood stream via the heart and lungs to the hepatic portal system where they feed on blood cells, grow, and develop into adolescent male and female worms which pair up and mature^4^. The couples remain *in copula* and migrate to the mesenteric venous plexus where they release ~ 300 eggs per day^5^. Many eggs fail the extravasation process required for expulsion in faeces and instead get swept away in the blood flow and entrapped in the liver^6^. Here, the lodged eggs release antigenic components that drive a strong inflammatory response, leading to the recruitment of multiple immune cells that lead the formation of granulomas and fibrotic sequelae around the eggs, which cause significant pathology and are a hallmark of schistosomiasis^7^. The significant worm and egg burdens in parasitised individuals in endemic countries cause substantial morbidity and, in many cases, lead to death. Chemotherapy relies on a single drug, praziquantel (PZQ), however, while PZQ helps limit the disease, high cure rates in a population are rare and concerns exist regarding the emergence of drug resistance^8^.

Protein kinases control most biological processes in organisms through phosphorylation of the proteome and their catalytic activity is therefore tightly regulated. These enzymes can autophosphorylate, switching themselves from an inactive to active conformation; they can then also phosphorylate downstream client proteins affecting cellular responses and behaviour^9^. Although the schistosome genome encodes 268 protein kinases^10,11^, few have been studied in detail, particularly in relation to schistosome form and function. The multifunctional Ca^2+^/calmodulin dependant protein kinase II (CaMKII) is a crucial transducer of Ca^2+^ signalling within cells and co-ordinates multiple processes in organisms ranging from synaptic plasticity, long-term potentiation and spatial memory through to ion channel regulation and cardiac signalling^12,13^. When Ca^2+^ levels rise, Ca^2+^ binds the Ca^2+^-sensing protein calmodulin (CaM) which then binds to the autoinhibitory regulatory segment of CaMKII and exposes the Thr^286^ residue (numbering for human CaMKIIα isoform) for autophosphorylation by a neighbouring CaMKII subunit, which in turn activates the kinase^14,15^. CaMKII can then remain activated even in the absence of Ca^2+^/CaM as the phosphorylation prevents the regulatory domain from rebinding the catalytic domain; therefore, CaMKII can acquire autonomous, Ca^2+^ independent, activity. Although the exact molecular target(s) for PZQ in schistosomes remain elusive, PZQ disrupts Ca^2+^ homeostasis in the worms. This effect is probably mediated via Ca^2+^ channels (Cavß) and/or transient receptor potential (TRP) channels^16–18^ among others. In adult *S. japonicum*, CaMKII gene expression was shown to increase following exposure to sub-lethal concentrations of PZQ, and suppression of CaMKII gene expression improved PZQ efficacy^19^. More recently, in *S. mansoni*, PZQ efficacy improved against normally refractory juvenile parasites when they were co-treated with non-selective CaMK inhibitors^20^.

Recently we undertook a deep phosphoproteomic analysis of *S. mansoni* adult worms and discovered that CaMKII phosphorylation motifs were highly represented in the dataset, highlighting the likely importance of this kinase in these worms^21^. Here we aimed to investigate in detail the *S. mansoni* CaMKII protein, functionally map the protein within human infective life stages of the parasite and determine the importance of CaMKII to *S. mansoni* homeostasis and survival.

## Results

### CaMKII in *S. mansoni*

**Figure 1:**
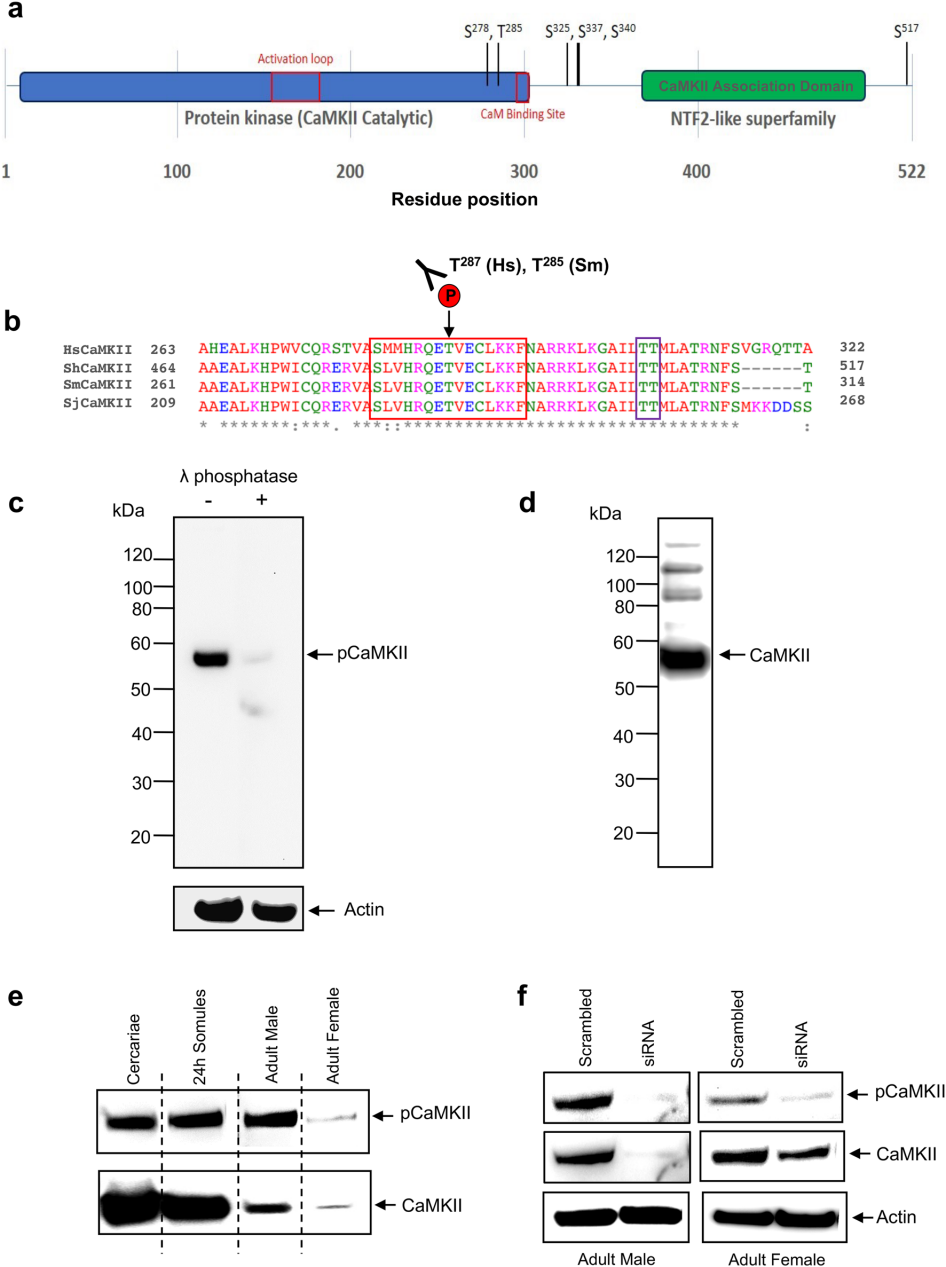
CaMKII in *Schistosoma mansoni*. (**a**) Schematic diagram highlighting the main features of the predicted *S. mansoni* CaMKII (Smp_011660.2) with confirmed phosphorylation sites^21^ indicated; further detail is provided in Supplementary Fig. 1. (**b**) Multiple Clustal Omega alignment of amino acid sequences spanning the anti-phospho CaMKII (Thr^286^) antibody recognition site for the *S. mansoni* CaMKII and relevant CaMKII sequences from human (HsCaMKIIβ; Q13554-2, Uniprot), *Schistosoma haematobium* (ShCaMKII; MS3_0019207.1, WormBase Parasite) and *Schistosoma japon*icum (SjCaMKII; EWB00_003065.2, WormBase Parasite). The antibodies only bind CaMKII when Thr^287^ (HsCaMKIIβ numbering) is phosphorylated and also typically recognise 5–7 residues either side of the phosphorylated site. (**c**, **d**) Schistosomula protein extracts (~ 1000 schistosomula) were processed for western blotting and probed with either anti-phospho CaMKII (Thr^286^) or anti-‘total’ CaMKII antibodies (abs); lambda phosphatase treatment was used to confirm that the anti-phospho abs reacted only with phosphorylated CaMKII and actin was used as an inter-lane loading control to demonstrate protein presence in the treated lane. (**e**) Protein extracts of different *S. mansoni* life stages (~ 1000 cercariae, ~ 1000 schistosomula, mature single male, mature single female) were processed for western blotting and probed with anti-phospho CaMKII (Thr^286^) or anti-‘total’ CaMKII abs; images shown are from different blots, separated by the dotted lines. (**f**) Adult male or female worms were electroporated with either scrambled (control) small interfering ribonucleic acid (siRNA) or CaMKII siRNA and were cultured for four days before proteins were extracted and processed for western blotting with either anti-phospho CaMKII (Thr^286^) or anti-‘total’ CaMKII antibodies; actin was used as an inter-lane loading control. The western blots are representative of those obtained in two independent experiments.

A single CaMKII gene predicted in the *S. mansoni* genome (WormBase ParaSite: Smp_011660) is responsible for three CaMKII isoforms (Smp_011660.1-3, 512, 522, and 401 a.a., respectively), that display typical CaMKII features (Fig. 1a; Supplementary Fig. 1). This CaMKII was most similar (77% identity) to human CaMKIIβ (Q13554-2) and strong similarly exists between the sequences of *S. mansoni*, *S. japonicum*, and *S. haematobium*. Analysis of our phosphoproteomic data^21^ reveals that the *S. mansoni* CaMKII is phosphorylated on at least six serine/threonine residues (Fig. 1a), including Thr^285^ that is conserved with Thr^286^ and Thr^287^ in human CaMKIIα and CaMKIIβ, respectively, the critical autophosphorylation site that leads to activation of the kinase^14^ (Fig. 1b). Two further threonine (Thr^305/306^) residues that when phosphorylated in humans result in autonomous CaMKII activity are also conserved (Thr^204/205^) in the *S. mansoni* protein (Fig. 1b), although these phosphorylation events were not captured in our phosphoproteomic screen^21^. Because *S. mansoni* CaMKII Thr^285^ is conserved and subject to activation-dependent phosphorylation, we selected a monoclonal anti-phospho CaMKIIα (Thr^286^) antibody that detects CaMKII only when phosphorylated (i.e. activated) (Fig. 1b) and initially screened it against *S. mansoni* schistosomula extracts. A single immunoreactive band was detected (~ 58 kDa), similar to the predicted MW of Smp_011660.2 (58.2 kDa), and pre-treatment of blots with lambda phosphatase substantially reduced immunoreactivity demonstrating that the antibody only detects the phosphorylated form of the kinase (Fig. 1c). Furthermore, a ‘total’ monoclonal anti-CaMKII antibody that recognises several CaMKII isoforms in humans irrespective of phosphorylation status detected a strongly immunoreactive protein of similar molecular weight in *S. mansoni*, and three other larger non-specific weakly immunoreactive proteins (Fig. 1d). This latter antibody is raised against a synthetic peptide surrounding Val^184^ of human CaMKIIα, a region also highly conserved in the *S. mansoni* homologue (Supplementary Fig. 1). Next, protein extracts were prepared from cercariae, 24 h in vitro cultured schistosomula, and mature adult male and female worms and processed for western blotting. Phosphorylated CaMKII was present in all these life stages with levels broadly corresponding to the expression pattern of the total protein (Fig. 1e). Finally, we performed RNA interference (RNAi). Mature adult worms electroporated with small interfering RNAs (siRNA) targeting Smp_011660.2 displayed a marked reduction in immunoreactivity with either the anti-phospho or anti- ‘total’ CaMKII antibodies when compared with worms electroporated with scrambled (negative) siRNA (Fig. 1f), supporting recognition of the kinase and attenuation of CaMKII expression through RNAi.

### Inhibition of CaMKII with KN93

**Figure 2:**
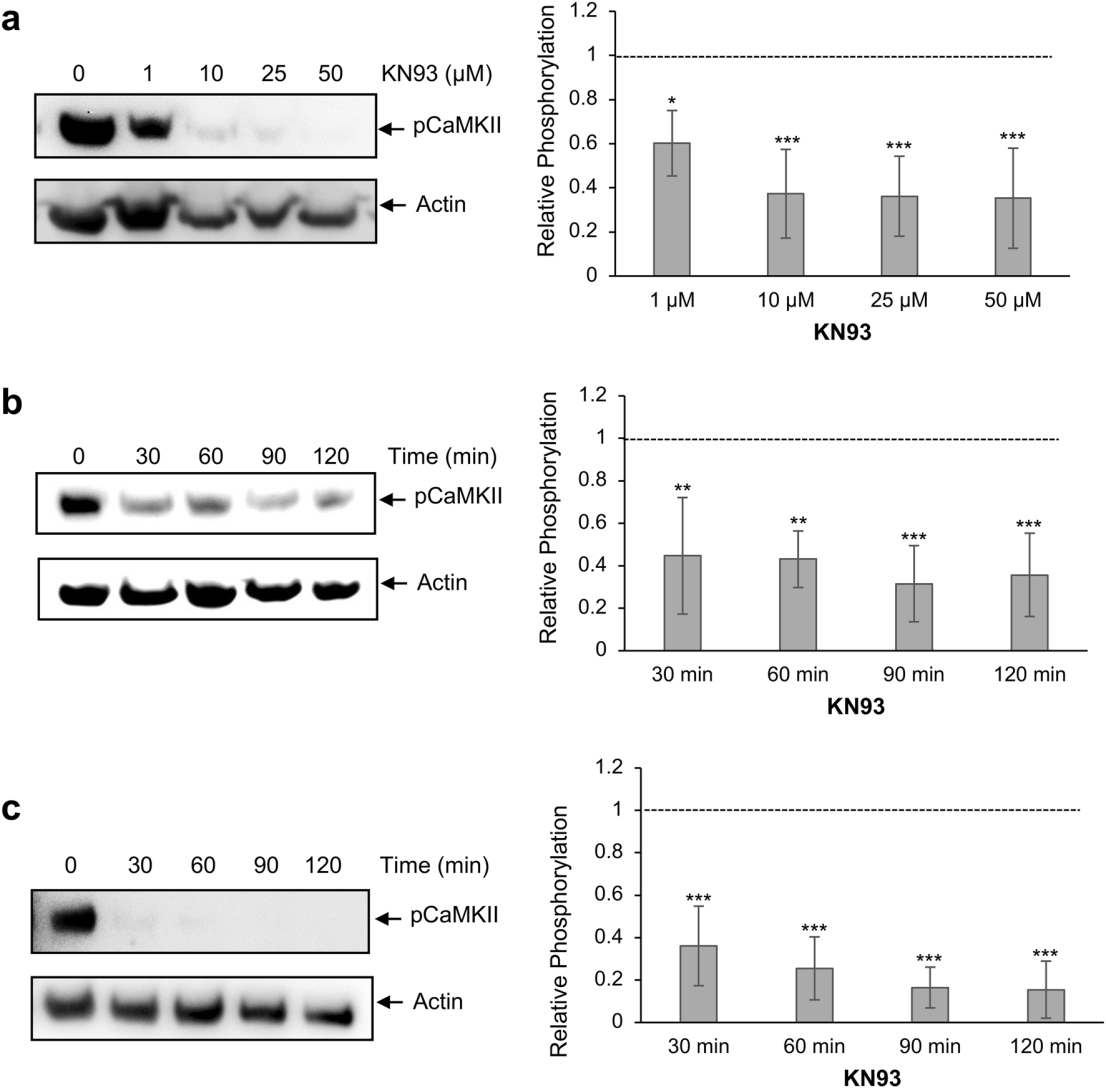
Inhibition of *Schistosoma mansoni* CaMKII phosphorylation with the CaMKII inhibitor, KN93. Either intact 24 h in vitro cultured schistosomula (~ 1000 per treatment) (**a**, **b**), or schistosomula homogenates (**c**), were exposed to (**a**) increasing concentrations of KN93 for 2 h, or (**b**, **c**) 10 µM KN93 for increasing durations, and the effects of KN93 on CaMKII phosphorylation evaluated by western blotting with anti-phospho CaMKII (Thr^286^) antibodies; actin was used as an inter-lane loading control. Representative blots are shown. Mean CaMKII phosphorylation levels (n = 4, ± SD; graphs) were determined and phosphorylation change calculated relative to the phosphorylation levels of controls (0) that were assigned a value of 1 (shown as dotted line); *p ≤ 0.05, **p ≤ 0.01, ***p ≤ 0.00 (ANOVA) when compared with control levels.

The CaMKII inhibitor KN93, which blocks CaMKII activation by binding to Ca^2+^/CaM, was next employed to assess its effects on *S. mansoni* CaMKII phosphorylation (activation). When 24 h in vitro cultured schistosomula were exposed to KN93 for 2 h, a significant reduction in CaMKII activation occurred with > 1 µM (*p* ≤ 0.05), with the greatest reduction of 65% seen at 50 µM (*p* ≤ 0.001) (Fig. 2a). When schistosomula were exposed to 10 µM KN93 for increasing durations, mean phosphorylation levels decreased with time with a maximal effect observed at 90 min (68% reduction; *p* ≤ 0.001) (Fig. 2b). Finally, the effects of KN93 were evaluated in disrupted schistosomula whereby parasites were homogenised in kinase buffer and 10 µM KN93 was added to each of the samples for increasing durations. Under these conditions, enhanced inhibition was observed with mean phosphorylation levels reduced by a striking 85% at 120 min (*p* ≤ 0.001; Fig. 2c). These data demonstrate that KN93 is an effective inhibitor of CaMKII phosphorylation/activation in *S. mansoni*, including in live worms, and further support the use of anti-phospho CaMKII antibodies for analysis of CaMKII activation profiles in schistosomes.

### The effect of praziquantel on *S. mansoni* CaMKII activation

**Figure 3:**
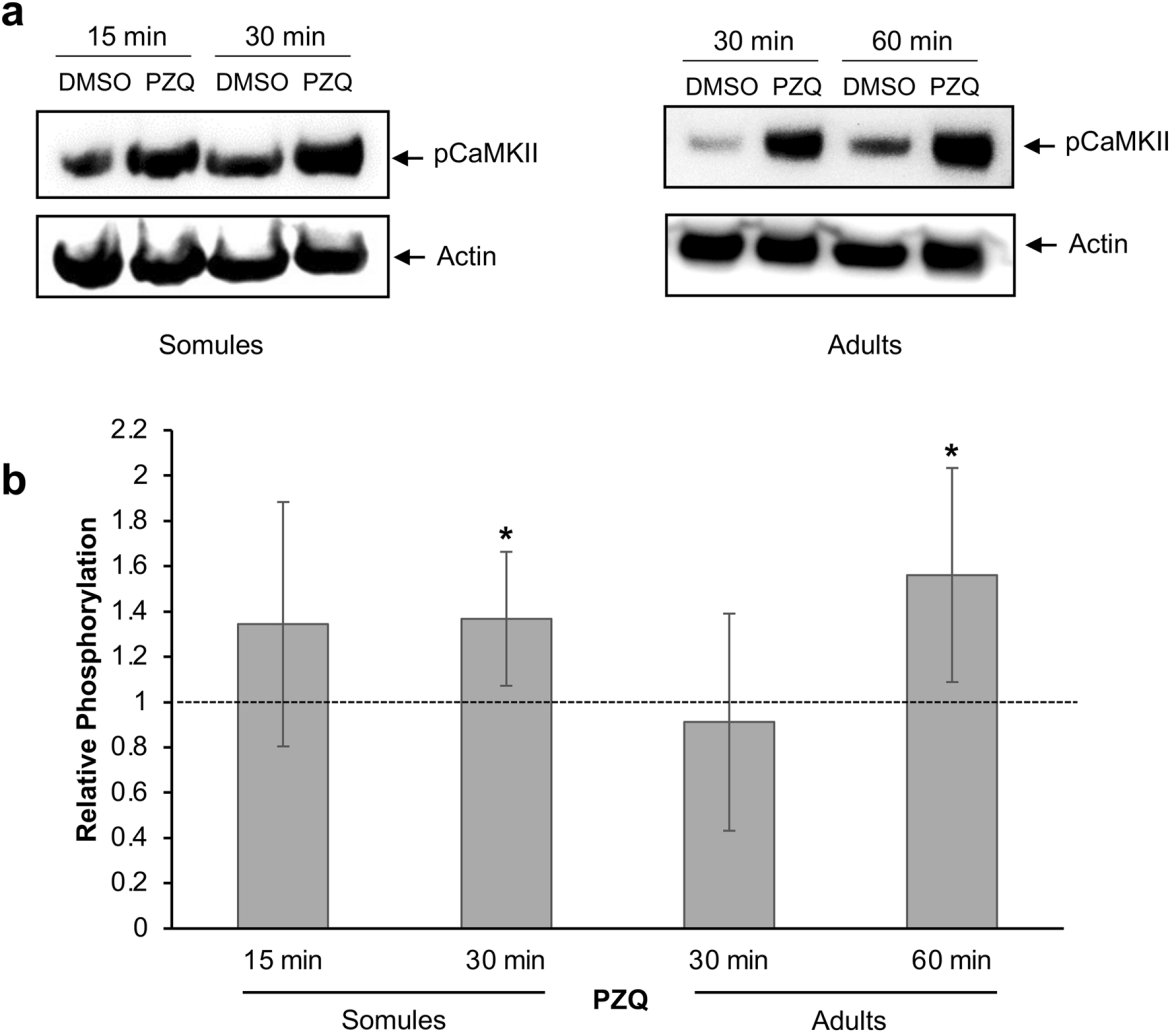
Praziquantel (PZQ) stimulates CaMKII phosphorylation in *Schistosoma mansoni* schistosomula and adults. (**a**) 24 h in vitro cultured schistosomula (~ 1000 per treatment), or (**b**) mixed male and female adult worms were exposed to PZQ (0.2 µg/ml) or DMSO (control) for increasing durations. Proteins were extracted and processed for western blotting with anti-phospho-CaMKII (Thr^286^) antibodies; actin was used as an inter-lane loading control. Representative blots are shown. Mean CaMKII phosphorylation levels (n = 4, ± SD; graphs) were determined and phosphorylation change calculated relative to the phosphorylation levels of DMSO controls that were assigned a value of 1 (shown as dotted line); *p ≤ 0.05 (ANOVA) when compared with control levels.

Given the link between PZQ exposure and loss of Ca^2+^ homeostasis in schistosomes^17,18^, the effect of PZQ on *S. mansoni* CaMKII activation was explored. Although 3, 7 and 14 day-old schistosomula appear to be refractory to PZQ, skin stage schistosomula display some susceptibility, with adult worms being particularly susceptible^22–24^. We employed 0.2 µg/ml PZQ, a low dose that we envisaged could potentially impact signalling without leading to rapid deterioration of the parasite^22,24,25^. Although relatively high basal levels of CaMKII activation were observed in schistosomula, exposure to PZQ moderately enhanced activation (by ~ 37%; *p* ≤ 0.05) after 30 min exposure (Fig. 3a). Next assays were performed on adult worms (mixed sex population) and, across several experiments, only 60 min PZQ treatment significantly affected CaMKII activation (with an increase of 56% observed; *p* ≤ 0.05), with the apparently positive effects at 30 min remaining inconclusive due to higher variability between treatments (Fig. 3b).

### Mapping activated CaMKII in *S. mansoni*

**Figure 4:**
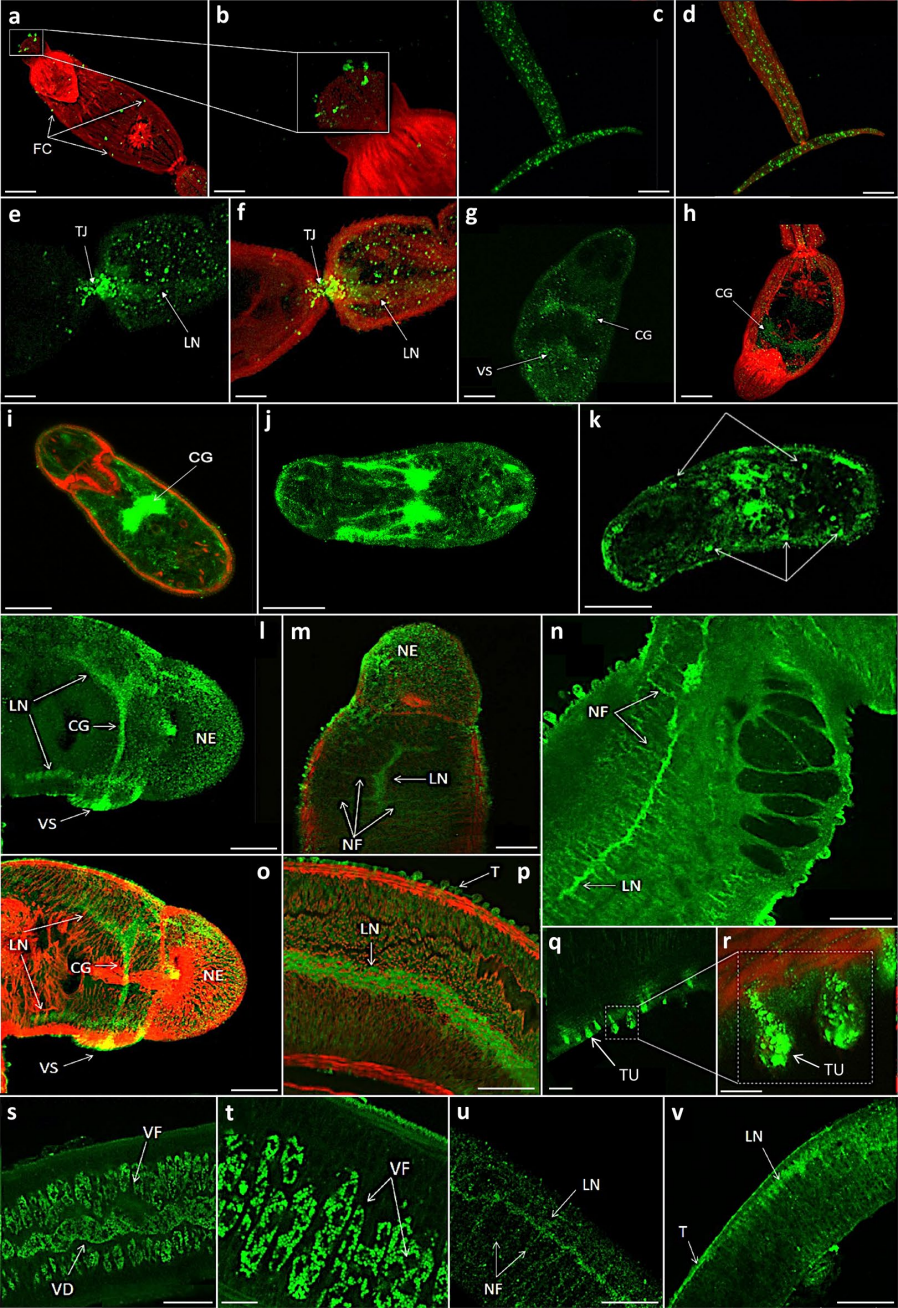
In situ immunolocalization of phosphorylated (activated) CaMKII in intact *Schistosoma mansoni* cercariae, schistosomula and adult worms. (**a**–**h**) Cercariae, (**i**–**k**) 24 h in vitro cultured schistosomula, (**l**–**r**) male, and (**s**–**v**) female worms were fixed and incubated with anti-phospho-CaMKII (Thr^286^) antibodies, followed by Alexa Fluor 488 secondary antibody (green); parasites were also stained with rhodamine phalloidin (red; shown in some images as overlays) to reveal actin filaments. Images are z-axis projections of either the whole parasite or of deep scans within the parasite displayed in maximum pixel brightness mode except for j, k, q, and r which are derived from single z-scans. Scale bar: b, e, f, r, t = 10 µm; a c, d, g–k, q, s = 25 µm; l–p, u, v = 50 µm. Abbreviations: CG, cephalic ganglion; FC, flame cells; LN, longitudinal nerve; NE, nerve endings; NF, nerve fibres; T, tegument; TJ, tail junction; TU, tubercles; VD, vitelline duct; VF, vitelline follicles; VS, ventral sucker.

We next performed mapping of phosphorylated CaMKII in intact cercariae, schistosomula and adult worms using our published approaches that employ confocal microscopy and immunofluorescence with anti-phospho antibodies to detect the activated kinase^26,27^. In all cases the negative controls displayed negligible fluorescence (Supplementary Fig. 2). In cercariae, activated CaMKII was prominently seen at the apical papillae, consistently displaying as six clusters of spots, with immunoreactivity also seen at distinct organised foci in the head that could represent sensory structures or flame cells (Fig. 4a, b). Activated CaMKII also localised to punctate regions of the muscular tail (Fig. 4c, d), the head–tail junction (Fig. 4e, f), and was also observed in the nervous system, including the longitudinal nerve, cephalic ganglia and the nerve net around the ventral sucker (Fig. 4e–h). In 24 h in vitro cultured schistosomula, activated CaMKII was prominently associated with the cephalic ganglia, longitudinal nerves and ventral sucker nerve net (Fig. 4i–j), and was also seen at the tegument (Fig. 4i) and punctate structures resembling those seen in the cercariae (Fig. 4k). In adult male worms, activated CaMKII was clearly observed along the full length of the longitudinal nerves, the cephalic ganglion, the delicate peripheral nerve networks and nerve endings including those of the oral and ventral suckers (Fig. 4l–p), the parenchyma, muscle, and the tegument and tubercles (Fig. 4p–r). Finally, in female worms, activated CaMKII associated with the vitellaria in which the specialised stem cells could be clearly seen (Fig. 4s, t); in addition, as for males, activated CaMKII was observed in the longitudinal nerves, nerve fibres, and tegument (Fig. 4u, v). Activated CaMKII did not appear in the male and female sexual organs including the ovaries, ootype and Mehlis’ gland complex of the females, or the testes and seminal receptacle complex of the males. Collectively, these results highlight that there is extensive CaMKII activation in the nervous system, tegument and vitellaria of *S. mansoni*.

### Effect of CaMKII RNAi on adult worm phenotype

**Figure 5:**
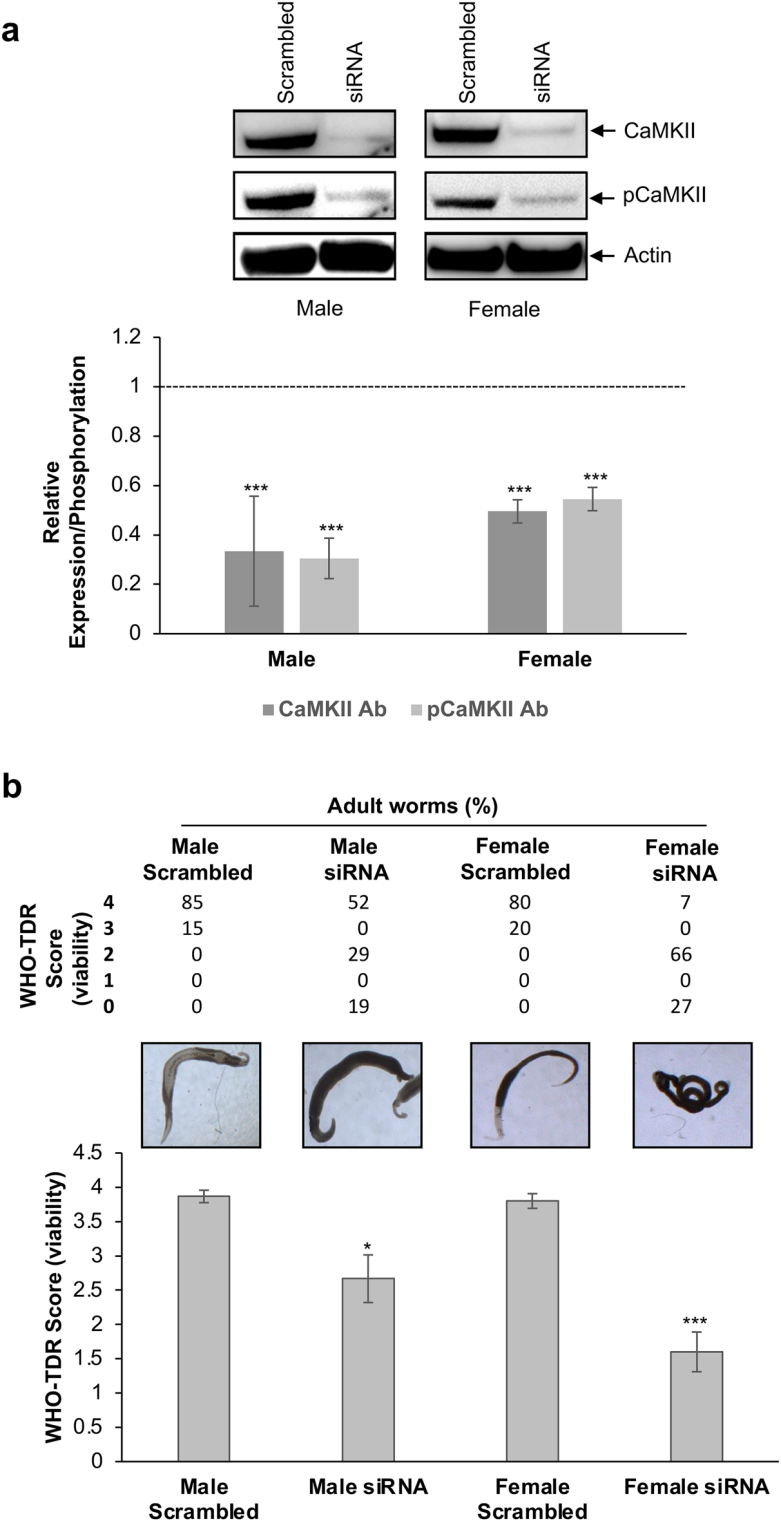
Suppression of CaMKII expression/activation in adult male and female *Schistosoma mansoni*, reduces worm movement and viability. (**a**) Adult worms were electroporated with either scrambled (control) small interfering ribonucleic acid (siRNA) or CaMKII siRNA and were cultured for four days before proteins were processed for western blotting with either anti-‘total’ CaMKII or anti-phospho CaMKII (Thr^286^) antibodies; actin was used as an inter-lane loading control. Representative blots are shown. Mean relative changes (n = 3, ± SD; graph) in CaMKII expression/phosphorylation were determined and calculated relative to the levels present in the scrambled controls that were assigned a value of 1 (shown as dotted line). (**b**) Adult worms were also assessed for the effects of siRNA treatment on movement (viability) using the WHO-TDR score index, where 4 = normally active and 0 = total absence of motility; micrographs show representative worms for each treatment. The mean viability score (n ≥ 15 per treatment, ± SD; graph) of worms in each treatment was calculated. *p ≤ 0.05, and ***p ≤ 0.001 (ANOVA) when compared with the scrambled (control) siRNA for each worm sex.

Because activated CaMKII strongly associated with the neuronal and tegumental systems of *S. mansoni*, we aimed to determine the effect of RNAi on adult worm movement and viability. Four days after electroporation with siRNA targeting CaMKII, a highly significant, 66 and 52% (*p* ≤ 0.001), attenuation of CaMKII expression occurred in adult male and female worms, respectively (Fig. 5a). Furthermore, the reduction in levels of phosphorylated (activated) CaMKII following siRNA (70 and 45% in male and female worms, respectively) were similar to that observed for the CaMKII protein itself, demonstrating that hyperactivation of remaining CaMKII protein does not occur as a result of CaMKII knockdown (Fig. 5a). Suppression of CaMKII in adult worms substantially blunted their movement and viability (Fig. 5b). Four days after siRNA, all male and female scrambled control worms survived and displayed normal movement (WHO-TDR score 4) or slowed activity (score 3), with ≥ 80% normal motility observed in the two groups. In contrast, 19% of male and 27% of female worms died following CaMKII siRNA, with 29% of males and 66% of females showing minimal (score 2) activity. Overall, activity levels of females dropped by a striking ~ 75% following CaMKII knockdown. Collation of TDR viability scores for worms within each treatment revealed that the overall viability score, linked to both movement and death, dropped by 31% (*p* ≤ 0.05) for males and 58% (*p* ≤ 0.001) for females.

### Network analysis of *S. mansoni* CaMKII protein–protein associations

**Figure 6:**
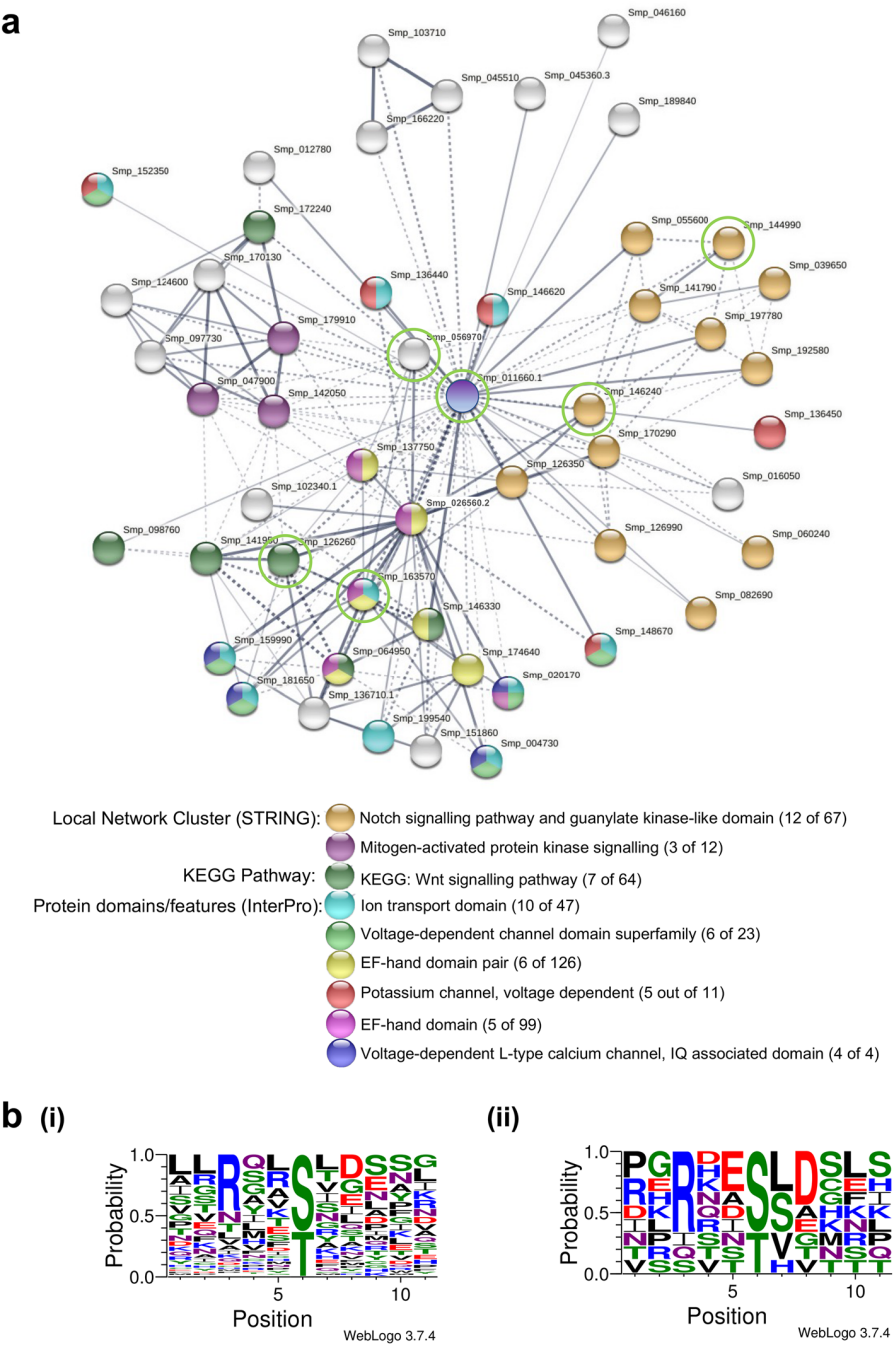
Network analysis of *Schistosoma mansoni* CaMKII associations. The CaMKII sequence (Smp_011660) was queried against the STRING database to identify putative physical/functional protein–protein associations. (**a**) First shell association map of medium confidence (STRING global score ≥ 0.40; protein–protein enrichment *p* < 1.0e^−16^) protein–protein (node) associations in confidence mode (thicker lines = higher confidence); inter-cluster edges are shown with dashed lines. Smp_011660 appears in the centre of the network. Functional enrichments were evaluated (Supplementary Table 1) and selected examples (see key) comprising local node clusters, KEGG pathways, and protein domain/features visualised and overlain in different node colours. The number of nodes shown for each enrichment is against the background of the entire *S. mansoni* genome as currently annotated within STRING. (**b**) Motif analyses generated with WebLogo 3 (probability mode) to illustrate (i) the nature of the putative CaMKII phosphorylation sites found in the association partners using NetworKIN, and (ii) those sites confirmed as being present in our phosphoproteomic dataset^21^, with (**a**) network proteins (nodes) highlighted for the latter with a green circle.

At medium confidence (STRING global score ≥ 0.40), 51 *S. mansoni* proteins were predicted to associate with CaMKII (Smp_011660) with 183 interactions (edges) present (Fig. 6a). Functional enrichment analysis of the network revealed 23 enriched local network clusters (STRING) (Supplementary Table 1), including notch signalling and mitogen-activated protein kinase (MAPK) signalling pathway components; in addition, the Wnt signalling pathway (KEGG) was enriched (Fig. 6a) as well as proteins for 5 other KEGG pathways (Supplementary Table 2). InterPro protein domain enrichments (43 in total) included ion transport domain, EF-hand domain, and voltage dependent channel containing proteins (Fig. 6a; Supplementary Table 3).

Potential CaMKII-mediated phosphorylation sites within the proteins present in the association network were next evaluated using NetworKIN. A total of 123 potential CaMKII phosphorylation sites (78 Ser/45 Thr) across 12 of the 51 proteins were identified (Supplementary Table 4; NetworKIN score > 5) and motif analysis revealed a preponderance of the CaMKII kinase consensus sequence (XRXXS/TX) (Fig. 6). A total of 9 phosphorylation sites within 6 network proteins from our global *S. mansoni* phosphoproteomic data set were then successfully mapped to these putative sites (Supplementary Table 5), supporting further the protein–protein association data; the putative CaMKII phosphorylation sites were confirmed in: Smp_011660, CaMKII; Smp_056970, glyceraldehyde-3-phosphate dehydrogenase; Smp_126260, protein phosphatase-2b; Smp_144990, amyloid beta A4 protein related; Smp_146240, discs large; and Smp_163570, ryanodine receptor related (Fig. 6a).

## Discussion

Through its unique ability to integrate a variety of Ca^2+^ signals into specific outcomes, CaMKII plays multiple fundamental roles in eukaryotes^28,29^; however, its importance to parasite homeostasis is poorly understood. CaMKII appears to be involved in proliferation of *Trypanosoma cruzi* epimastigotes^30^, and CaMKII transcription was elevated in *S. japonicum*^19^ after PZQ exposure highlighting a possible link between this drug and kinase in schistosomes. Here, we validated ‘smart’ anti-phospho CaMKII (Thr^286^) antibodies enabling the detection of exclusively activated CaMKII in *S. mansoni* and employed them to investigate CaMKII activation by PZQ and functionally localise CaMKII in cercariae, schistosomula and adult worms. In addition, we revealed that siRNA-mediated suppression of CaMKII activation (and expression) results in reduced motility and vitality of the mature adults.

Of the human CaMKII isoforms, *S. mansoni* CaMKII is most like human CaMKIIβ. The crucial autophosphorylation site (Thr^285^ in *S. mansoni* CaMKII) is present and the antibody target region (~ 15 a.a. around the phosphorylation site) is also highly conserved. The antibody reacts with a protein of similar MW to the predicted CaMKII, and both the removal of phosphate by lambda phosphatase and treatment with KN93, which blocks CaMKII activation^31^, abolished or reduced immunoreactivity. Collectively, these data support that the anti-phospho CaMKII (Thr^286^) antibodies specifically detect the phosphorylated (activated) form CaMKII in *S. mansoni*. Activated CaMKII was detected in all life stages investigated.

Using confocal laser scanning microscopy, we mapped the functionally activated form of CaMKII within *S. mansoni* and discovered activated CaMKII predominantly in the nervous system of cercariae, schistosomula and adult male and female worms. Activation at the longitudinal nerves was prominent, with further CaMKII activation seen in the nerve projections, particularly in the adults, culminating in nerve endings towards the surface of the parasite. In cercariae and schistosomula, there was also a significant association of activated CaMKII in the cephalic ganglion, the nerve centre of the parasite. Collectively, these results are intriguing as research in other organisms (e.g. *Xenopus, M. musculus* and *H. sapiens*) has revealed CaMKII to be involved in the regulation of neuronal development, synaptic plasticity, learning and memory^28,32^. In *Caenorhabditis elegans*, the CaMKII homologue UNC-43 has been found to regulate synaptic strength^33^, control muscle excitation and worm locomotory activity^34,35^, and regulate lifespan through the DAF16 FOXO transcription factor orthologue^36^. The neuronal localisation of CaMKII seen in adult *S. mansoni* was also similar to that observed for protein kinase A (PKA), which was found to regulate neuromuscular activity of the male and female worms^37^. Activated CaMKII was also prominent at the tegument of adult worms particularly within the tubercles of males. This finding opens the possibility that CaMKII functional responses could be regulated by host molecules, such as insulin and epidermal growth factor, which could bind receptors at the parasite surface and alter worm behaviour. In this context, we have previously shown such factors to activate protein kinase C (PKC) and extracellular signal-regulated kinase (ERK) in *S. mansoni*^27,38^. Although activated CaMKII was not evident within the testes of males or in the ovaries of females, it was observed in the female vitellaria, specialised tissues unique to flatworms, where it was prominent in the individual vitellocytes within the vitelline follicles. The vitellocytes exist as stages one to four, with stage one being the progenitor cell and four being the mature, terminally differentiated cell which possesses ribosomal complexes and large vitelline and lipid droplets that amalgamate forming the eggshell. Thirty to forty of these S4 cells merge with an oocyte in the vitello-oviduct and migrate to the ootype where egg biogenesis ensues^39^. The role of CaMKII in vitellocyte function remains unknown but is worthy of further investigation considering the importance of vitellocytes to egg production and thus disease progression and schistosomiasis transmission.

Even though PZQ has been used for decades to control human schistosomiasis the molecular target of this drug remains poorly defined and is the focus of much research and speculation, possibly because multiple targets exist. Exposure of schistosomes to PZQ evokes fast Ca^*2*+^ influx into the worm, tegument damage and muscle paralysis. Proteins, including a voltage-gated Ca^2+^ channel (Cavß)^40^, an ATP-binding cassette (ABC) transporter^41^, a serotoninergic G-protein-coupled receptor^42^, and a TRP channel have been implicated in the PZQ response^43^. Here, we show that low dose (0.2 µg/ml) PZQ exposure enhanced CaMKII phosphorylation (activation) in 24 h schistosomula and adult worms, likely mediated by calcium influx and calmodulin binding. Protein kinases such as CaMKII phosphorylate multiple cellular targets including receptors and ion-channels^44,45^ altering conformation, protein–protein binding, and target protein activity. Therefore, our finding that PZQ activates CaMKII highlights a need to consider PZQ response mechanisms broadly and, where possible, integrate the concept of cellular signalling/protein interactions into putative PZQ target characterisation/function. The *S. mansoni* regulatory myosin light chain (SmMLC) was also found to be a major binding partner of PZQ, suggesting that PZQ could mediate its effect on the parasite through modifying SmMLC function^46^. Interestingly, SmMLC was also phosphorylated in schistosomula upon exposure to PZQ^46^ with temporal kinetics similar to the CaMKII activation seen here. Because MLC is a potential target of CaMKII in muscle alongside myosin light chain kinase^47^, CaMKII could potentially cause PZQ-mediated SmMLC phosphorylation in schistosomes. Further work is required to unravel the modulatory effects of PZQ on CaMKII and its relevant functional partners. Such investigations could include separate sex experiments with adult worms of different ages, with and without human serum, because males are more susceptible than females and worm susceptibility changes with age^16^, and because while the tegument damage occurs in both sexes, human serum seems to mitigate the effects of PZQ in females^48^. Studies with PZQ enantiomers and anti-schistosome PZQ derivatives^49^ would also highlight further the importance CaMKII in schistosome PZQ response mechanisms.

When CaMKII gene transcription was suppressed using RNAi in adult *S. japonicum* by 50–69%, the suppressive effect of an IC_50_ dose of PZQ on worm movement was enhanced over 72 h when compared to PZQ alone^19^, leading the authors to suggest that CaMKII mitigates the effect of PZQ, possibly by stabilising Ca^2+^ fluxes within the parasite muscle and tegument. However, no detrimental motility effects were seen following RNAi knockdown in the absence of PZQ^19^. Here, we employed siRNA to supress CaMKII expression in *S. mansoni* adults and demonstrate, through western blotting with antibodies against total CaMKII and phosphorylated CaMKII, a highly significant attenuation of both CaMKII protein and CaMKII protein activation in both sexes. Moreover, we reveal that four days after siRNA treatment, 19% of male worms and 27% of female worms died in comparison to none in the scrambled controls; the surviving male and female worms displayed significantly attenuated motility, with the effect in the females more pronounced. Collectively, these data show that the CaMKII protein is essential not only for adult worm movement but also for worm survival. Given this finding, future investigations employing RNAi to supress CaMKII expression in skin schistosomules would be valuable, enabling the role of CaMKII in growth and survival of developing worms to be determined. The effects of CaMKII protein knockdown on adult worm motor activity can be explained by the presence of the activated enzyme in the nervous system and musculature of the parasite observed by confocal laser scanning microscopy, further supported by single cell transcriptome data for the CaMKII gene Smp_011660 obtained from the SchistoCyte Atlas (https://www.collinslab.org/schistocyte/)^50^ (Supplementary Fig. 3). The mechanisms governing CaMKII-mediated movement in schistosomes might be similar to those for UNC-43 in *C. elegans*, although the latter are not well elucidated. Schistosomes have been found to have unusual ‘hybrid’ musculature comprising striated muscle-like myosin filaments and smooth muscle-like actin filaments within a smooth muscle-like architecture, the activity of which is probably regulated by myosin phosphorylation^51^, possibly via CaMKII which can regulate smooth muscle contractility through complex mechanisms^29^. The mechanisms underlying the death of male and female adult worms following CaMKII knockdown require investigation, but they could be linked to the high levels of CaMKII activity seen within the nervous system of *S. mansoni* given that CaMKII dependent signalling in neurons has recently been found to be essential for mouse survival^52^.

To obtain insight into potential CaMKII protein–protein associations in schistosomes we interrogated the STRING database for Smp_011660. A total of 51 medium confidence proteins were mapped to the network with 183 first shell interactions identified. Of these 51 proteins, 12 were predicted by NetwroKIN to harbour 123 potential CaMKII phosphorylation sites and nine of these were mapped to our recently published phosphoproteome data^21^, identifying them as bona fide target sites. One of these sites was in a ryanodine receptor (RyR)-related protein and such receptors regulate intracellular Ca^2+^ release from the endoplasmic/sarcoplasmic reticulum in excitable nerve and muscle cells^53^; in *C. elegans*, the RyR homologue UNC-68 regulates body wall muscle contraction^54,55^, linking CaMKII to such processes in schistosomes. Gene ontology analysis of the STRING network revealed many functionally enriched network clusters and KEGG pathways, such as Notch, Wnt and MAPK pathways as well as numerous enriched protein domains including ion-transport and voltage-dependent channel protein domains, highlighting CaMKII as a putative central regulator of multiple fundamental mechanisms in schistosomes. For example, Smp_026510 (MEK2) is a putative target of CaMKII and this protein is an upstream regulator of ERK, a protein that we found to be phosphorylated by PZQ exposure and to profoundly affect *S. mansoni* motility, pairing, attachment, and egg release^38^. Moreover, the notch pathway has been found to regulate oogenesis and embryogenesis in *S. mansoni*^56^. Given that Wnt and notch are central to organism development, it is possible that CaMKII interactions with MAPK signalling/ion channels/other components identified in the network underpin the effects of CaMKII protein knockdown on adult worm vitality. Therefore, in addition to further revealing CaMKII as an active player in the PZQ-mediated schistosome response, our finding that CaMKII as an essential element to schistosome survival opens opportunities for the development of novel CaMKII-based schistocidal therapeutics.

## Methods

### Parasites

*Biomphalaria glabrata* snails, infected with *S. mansoni* (Puerto Rican strain), were provided by the Biomedical Research Institute (BRI; Maryland, USA) via the National Institute of Health–National Institute of Allergy and Infectious Disease (NIH-NIAID) Schistosomiasis Resource Center under NIH-NIAID Contract No. HHSN272201000005I, or by Nuha Mansour/Quentin Bickle from the London School of Hygiene and Tropical Medicine (LSHTM, UK). Snails were maintained at 26 °C under a 12 h/12 h light dark cycle in boxes containing tap water filtered through a Brimak carbon filtration unit (Silverline UK; Devon, UK). When patent, snails were placed under a light source and the emergent cercariae were collected. They were then either processed for immunohistochemistry or transformed mechanically into schistosomula using our standard approaches^57,58^. The transformed schistosomula were next loaded into 48-well tissue culture plates (Nunc) (~ 1000 schistosomula/ml/well) in Basal Medium Eagle (BME) containing antibiotics/antimycotics (Sigma) and were incubated at 5% CO_2_/37 °C for 18–24 h before experimentation.

Adult worms were obtained from BioGlab Ltd (courtesy of Professor Mike Doenhoff, the University of Nottingham, UK), where laboratory animal use was within a designated facility complying with the UK Animals (Scientific Procedures) Act 1986; the University of Nottingham Ethical Review Committee approved work involving mice and work was carried out under Home Office licence 40/3595. Upon receipt, the perfused adult worms were placed in Roswell Park Memorial Institute (RPMI) medium containing antibiotics/antimycotics and were equilibrated for 2 h (5% CO_2_/37 °C) before use.

### Bioinformatics

Protein sequences for the four human isoforms of CaMKII, α, β, γ and δ, were obtained from Uniprot (https://www.uniprot.org/) (CaMKIIα accession number, Q9UQM7; β, Q13554; γ, Q13555; δ, Q13557). A protein basic local alignment search tool (pBLAST) query was performed at NCBI (https://blast.ncbi.nlm.nih.gov/) and WormBase Parasite (https://parasite.wormbase.org/index.html) to identify *S. mansoni* CaMKII-like proteins; this process was then repeated to find *S. japonicum* and *S. haematobium* homologues. Protein sequence alignments were generated using Clustal Omega https://www.ebi.ac.uk/Tools/msa/clustalo/). The binding sites of the anti-phospho CaMKII antibodies were obtained from PhosphoSitePlus (https://www.phosphosite.org/). The sites of phosphorylated residues within CaMKII were identified using our published phosphoproteomic data for *S. mansoni*^21^ and CaMKII protein domains were predicted by the NCBI Conserved Protein Domain tool (https://www.ncbi.nlm.nih.gov/Structure/cdd/).

Putative associations between *S. mansoni* CaMKII (Smp_01660.1) and other S*. mansoni* proteins were queried using Search Tool for Retrieval of Interacting Genes (STRING; version 11.0)^59^ in ‘protein’ mode and the association map was visualised at medium (STRING global score ≥ 0.40) confidence. Functional enrichments were evaluated within STRING and overlain onto the association map to highlight protein associations of interest, with a particular focus on domain enrichment within the map. Potential CaMKII-mediated phosphorylation sites within the putative interacting proteins were interrogated using KinomeXplorer, NetworKIN^60^ (minimum score 5; 1 prediction per site). These were then mapped on to phosphorylation site data generated in our phosphoproteomic screen^21^ to permit identification of definitive phosphorylation events within the network likely driven by CaMKII. Sequence logos were generated in probability mode using WebLogo3 (http://weblogo.threeplusone.com/create.cgi).

### Detection of CaMKII/CaMKII phosphorylation by western blotting and effects of inhibitors and praziquantel

Cercariae (~ 1000) or schistosomula (~ 1000) were pelleted by pulse centrifugation (~ 30 s) and lysed in hot LDS sample buffer (Invitrogen). Adult worms were homogenised on ice, using a motorised microfuge tube pestle (Kimble-Chase), in microfuge tubes containing radioimmunoprecipitation (RIPA) buffer (Cell Signalling Technology, CST; 20 µl/worm) and Halt protease/phosphatase inhibitors (Thermo Scientific); LDS sample buffer was then added. All lysates were heated to 95 °C for 5 min and briefly sonicated in a bath sonicator before electrophoresis or storage at − 20 °C.

Proteins were loaded onto Bolt Bis–Tris Plus gels, electrophoresed in a MES/SDS buffer system (Life Technologies), and transferred to nitrocellulose membranes. Blots were then blocked with 1% BSA in Tween 20 tris-buffered saline (TTBS; 1 h) and were incubated in anti-phospho-CaMKII Thr^286^ (CST; #12716) or anti-CaMKII (CST; #4436) rabbit monoclonal antibodies (1/1000) overnight at 4 °C. After washing (TTBS, three times 5 min each), blots were incubated in horseradish peroxidase (HRP)-conjugated secondary antibodies (1/5000) for 2 h and bands visualised using either ECL Prime (GE Healthcare) or West Pico (Thermo Scientific) substrate using a GeneGnome blot imager (Syngene). For re-probing, the nitrocellulose membranes were immersed in Restore Western Blot Stripping Buffer (Thermo Scientific) and rinsed with TTBS before incubating in primary antibodies. Actin, as loading control, was detected using HRP-conjugated anti-actin antibodies (Santa Cruz Biotechnology; 1:3000, 3 h). Intensities of immunoreactive bands were quantified using GeneTools (Syngene) and expression/phosphorylation levels normalised to actin. To dephosphorylated CaMKII, blots were immersed in a solution (1% BSA, 2 mM MgCl_2_) containing lambda phosphatase (400 U/ml) for 4 h. Blots were sometimes cut into smaller sections after Ponceau S staining to conserve antibody and fuller (uncropped) blot images are provided in Supplementary Figs. 4–6.

Live in vitro cultured schistosomula (24 h; ~ 1000/treatment) in BME and antibiotics/antimycotics were exposed to KN93 (Calbiochem #422,711) at increasing concentrations (1–50 µM, 2 h) and for different durations (30–120 min; 10 µM), with water (vehicle) controls; incubations were performed at 5% CO_2_/37 °C. After treatment, schistosomula were chilled rapidly on ice and processed for western blotting. In addition, KN93 assays were performed with schistosomula homogenates at room temperature. For this, 5000 schistosomula were pelleted by pulse centrifugation (30 s) and were homogenised using a glass homogeniser in 400 µl kinase buffer (CST) containing HALT protease/phosphatase inhibitor cocktail. The homogenate was divided equally into five microfuge tubes and 10 µM KN93 (or water control) added to each for increasing durations (30–120 min); homogenates were then processed for western blotting. Finally, live schistosomula (~ 1000) in BME, or adult worms in RPMI (one male, one female), were exposed to PZQ (Sigma; 0.2 µg/ml) or vehicle (DMSO, 0.01%) for increasing durations (15–60 min). The adult worms or schistosomula were then chilled rapidly on ice and processed for western blotting with anti-phospho CaMKII Thr^286^ antibodies.

### Immunofluorescence

Immunofluorescence was performed on intact parasites using our published protocols^3,37,38^ Briefly, cercariae, schistosomula, or adult worms were fixed in 3.7% paraformaldehyde (~ 4 h), blocked in 1% glycine (15 min), and permeabilized in Triton X-100/PBS (1 h) before washing in PBS and blocking in 10% normal goat serum (2 h; Thermo Fisher Scientific). Parasites were then incubated with anti-phospho-CaMKII (Thr^286^) antibodies (1:50 in 1% BSA), or 1% BSA alone (negative control), for 3 days at 4 °C with gentle agitation before extensive washing (three times for 30 min in PBS) and incubation in AlexaFluor 488 antibodies (1:500 in PBS)/rhodamine phalloidin (2 days). After final washing in PBS, parasites were mounted on slides (using silane prep slides (Sigma) for cercariae and schistosomula) in Vectashield (Vector Laboratories) and visualised using a Leica TCS SP2 AOBS confocal laser scanning microscope using 40 × or 60 × oil immersion objectives and images captured.

### Ribonucleic acid interference and phenotyping assay

Perfused adult worms were cultured overnight (5% CO_2_/37 °C) in wells of a 48-well tissue culture plate (Nunc; three males or females per well) each containing 1 ml of Opti-MEM, 2% antibiotics/antimycotics, and 4% foetal bovine serum (FBS). Adult worms were treated with a pool of 27-mer synthetic small interfering RNAs (siRNAs; Integrated DNA Technologies), specific for *S. mansoni* CaMKII, designed using the Custom RNAi Design Tool (IDT). Target sequences were: SmCaMKII siRNA1, 5’-CCCUGAAGCUAAGAAUUUAAUCAA-3’ spanning bp 765–790 of the CaMKII coding region RNA; SmCaMKII siRNA2, 5’-AGACUUACUCAGUUUCUAGAUAAAT-3’ spanning bp 1388–1413; and SmCaMKII siRNA3, 5’-GAAUUACUGUCAUCAGAAUAAUATT-3’ mapping to bp 412–437. The negative control “DS Scrambled Neg” siRNA (IDT; 5-CTTCCTCTCTTTCTCTCCCTTGTGA-3’) does not match any *S. mansoni* sequence. Adult worms were electroporated (square-wave, 20 ms pulse/125 V) in 4 mm electroporation cuvettes (VWR) containing 100 µl Opti-MEM and either 7.5 µg of DS Scrambled Neg, or 2.5 µg of each of the three siRNAs using a Gene Pulser Xcell (BioRad)^61^, before being maintained in Opti-MEM (with 2% antibiotics/antimycotics, 4% FBS; 5% CO_2_/37 °C) for four days.

Phenotype analysis was then performed by capturing 30 s movies of adult worms using a Motic SMZ-171 microscope equipped with a Moticam 1080 digital camera system. Each movie was viewed, and worm motility analysed and assigned a score according to the WHO-TDR scoring system^62^: 4 = normally active; 3 = slowed activity; 2 = minimal activity, occasional movement of head and tail; 1 = absence of motility apart from gut movement; 0 = total absence of motility. Finally, worms were processed for western blotting.

### Statistical analysis

The quantitative data from western blotting experiments and worm viability assays were analysed using one-way ANOVA and Tukey post-hoc multiple comparison with SPSS.

## Supplementary Information

Supplementary Information.

## Data Availability

All data generated or analysed during this study are included in the published article and its supplementary information files.
