# Supplementary material for: CaMKII regulates neuromuscular activity and survival of the human blood fluke Schistosoma mansoni
Source: Sci Rep. 2022 Nov 18;12:19831. doi: 10.1038/s41598-022-23962-8 (PMC9674609; doi:10.1038/s41598-022-23962-8)
Supplement: Supplementary file 1 — Supplementary Information. [file 41598_2022_23962_MOESM1_ESM.docx]

**Supplementary information**

**Supplementary Figure 1.** Multiple Clustal Omega sequence alignment of the *Schistosoma mansoni* (Sm) CaMKII protein (Smp_011660.2, WormBase Parasite) against Human (Hs) CaMKIIβ (Q13554-2, Uniprot), *Schistosoma haematobium* (Sh) CaMKII (MS3_0019207.1, WormBase Parasite) and *Schistosoma japonicum* (Sj) CaMKII (EWB00_003065.2, WormBase Parasite). The CaMKII catalytic domain (orange) and CaMKII association domain (NTF2-like; blue) are highlighted with open dashed boxes, confirmed phosphorylation sites ^21^ with red shaded boxes, antibody recognition sites with blue shaded boxes (with Val^184^ of human CaMKIIα in green), and activation loop and CaM binding site highlighted with yellow and salmon-coloured boxes respectively. The sequence length comprising the antibody recognition site is not publicly available and is approximated here based on the general approaches used to generate anti-peptide antibodies. The currently predicted ShCaMKII (MS3_0019207.1) has an N-terminal extension and a C-terminal deletion (with no apparent CaMKII association domain), skewing the alignment in these regions.

**Supplementary Figure 2.** Negative controls for *S. mansoni* immunofluorescence. Samples of (**a**) cercariae, (**b**) 24 h *in vitro* cultured schistosomula, and (**e-f**) adult female and male worms were processed for immunofluorescence with AlexaFluor 488 (green) and rhodamine phalloidin (red; to stain filamentous actin), but without anti-phospho-CaMKII (Thr^286^) antibodies. Images are z-axis projections displayed in maximum pixel brightness mode; those for the adult worms show (**e-f**) female anterior head region, (**g-h**) male mid region, and (**i-j**) male posterior tail region.

**Supplementary Figure 3.** Gene expression of CaMKII (Smp_011660) in *S. mansoni* mature adult worms. UMAP plots of (**a**) all cells from male and female worms, and (**b**) cells from male and female worms separately. Cell clusters highlighted (31 for nerve cells, N1-N33; 7 for muscle, M1- M7) were derived and adapted from single cell RNA-seq data available at SchistoCyte Atlas (https://www.collinslab.org/schistocyte/)^48^ queried for Smp_01160. Unlabelled clusters illustrate CaMKII expression across the non-nerve and muscle tissues.

**Supplementary Table 1.** Functional enrichment of *S. mansoni* CaMKII protein-protein association partners, showing local network clusters derived from STRING.

**Supplementary Table 2.** Functional enrichment of *S. mansoni* CaMKII protein-protein association partners, showing KEGG pathways derived from STRING.

**Supplementary Table 3.** Functional enrichment of *S. mansoni* CaMKII protein-protein association partners, showing InterPro protein domains and features derived from STRING.

**Supplementary Table 4.** Phosphorylation site analysis of *S. mansoni* CaMKII protein-protein association partners, showing NetworKIN predictions of putative CaMKII phosphorylation sites within substrate proteins (NetworKIN score greater than 5).

**Supplementary Table 5.** Phosphorylation site analysis of CaMKII protein-protein association partners, showing predicted sites from NetworKIN validated in Hirst *et al* [5].

**Supplementary Figure 4.** Images of western blots for Fig. 1 prior to cropping. The regions highlighted by the red boxes are those presented in the main manuscript.

**Supplementary Figure 5.** Images of western blots for Figs. 2 and 3 prior to cropping. The regions highlighted by the red boxes are those presented in the main manuscript.

**Supplementary Figure 6.** Images of western blots for Fig. 5 prior to cropping. The regions highlighted by the red boxes are those presented in the main manuscript.
